# The impact of the COVID-19 pandemic on self-efficacy scores and intensity of depressiveness in people aged over 60 years providing kinship foster care

**DOI:** 10.1186/s12877-023-03894-2

**Published:** 2023-03-30

**Authors:** Marta Giezek, Marek Landowski, Marta Kożybska, Beata Karakiewicz

**Affiliations:** 1 Subdepartment of Social Medicine and Public Health, Department of Social Medicine, Pomeranian Medical University in Szczecin, ul. Żołnierska 48, Szczecin, 71-210 Poland; 2 Department of Computer Science, Faculty of Computer Science and Telecommunications, Maritime University of Szczecin, Wały Chrobrego 1-2, Szczecin, 70-500 Poland; 3 Subdepartment of Medical Law, Department of Social Medicine, Pomeranian Medical University in Szczecin, ul. Żołnierska 48, Szczecin, 71-210 Poland

**Keywords:** Foster family, COVID-19 pandemic, Self-efficacy, Depression, Old age

## Abstract

**Background:**

The objective of this study has been to investigate whether the COVID-19 pandemic has had impact on self-efficacy scores and intensity of depressive severity in people aged over 60 who provide kinship foster care to their grandchildren.

**Methods:**

The study subjects were selected from among individuals aged over 60 years providing kinship foster care to their grand-children. The participants were asked to complete the Generalised Self-Efficacy Scale (GSE) and the Geriatric Depression Scale (GDS) before and during the pandemic. The questionnaire was com-pleted in whole twice by 40 participants.

**Results:**

There are no statistically significant differences between the GSE and GDS scores obtained before and during the pandemic. In study subjects with the oldest child in foster care aged 10 years or less, the GDS score decreased in a statistically sig-nificant way (p = 0.03). The correlation coefficient of the GSE and GDS scores before the pandemic was − 0.46 (p = 0.003), while that of scores during the pandemic was − 0.43 (p = 0.006).

**Conclusions:**

Neither the sense of self-efficacy nor the intensity of depressiveness of the study subjects changed significantly during the pandemic. Both before and during the pandemic, the increase in depressiveness was associated with a decrease in self-efficacy.

## Background

Foster parenting poses a particular challenge to the caregivers, involving, as it does, primarily undertaking and fulfilling the specific tasks of an adult in a relationship of protection and care of another individual. This study, which focuses on kinship foster care, concerns several problems of key contemporary health and psychosocial importance.

Undoubtedly, the most difficult task for those who decide to assume the role of a “foster parent” is not only to achieve balance between “taking care of the charges and taking care of oneself” [1], but also to understand the symmetry that occurs in this process. The quality of parenthood, including foster parenthood, is influenced both by external macrosocial transformations, such as historical events as well as demographic, health, cultural and social changes, and internal microsocial transformations, taking place within the family structure or in the local environment [2].

When old age is combined with role of grandparents and parents at the same time, as well as the fulfilment of duties towards grandchildren during the pandemic, the level of psychological well-being and the sense of self-efficacy of foster caregivers becomes an important aspect.

People aged over 60 years acting as foster caregivers for their grandchildren are in a particularly difficult situation, not only because of the need to combine the roles of grandparents parents, a situation that may cause conflict with the child’s biological parents, as well as educational, financial and organisational problems, [3] but also because these people are a group at increased risk of depression and COVID-19.

### Depression

Late-life depression (LLD) is most commonly understood as a heterogeneous affective disorder affecting people aged over 60 years. [4] According to the estimates of the World Health Organization (WHO), around 350 million people worldwide suffer from depression [5]. The PolSenior study has shown that the prevalence of mood disorders increases with age: in Poland they affect every fifth person aged 55–59, every fourth person aged 65–79 and every third person aged 80 or more [6]. For comparison, a study conducted in the United States among people aged over 60 years shows a prevalence of depressive symptoms at 9.8% [7]. Statistics also show that the number of people aged 50–60 suffering from depression is higher for women. These differences between men and women disappear at around the age of 80 [8].

### SARS-CoV-2 infections

Early reports worldwide suggest that the course of infection associated with the new strain of SARS-CoV-2 (Severe acute respiratory syndrome coronavirus), which causes a disease called COVID-19 [9], poses a particular risk for the elderly and patients with multiple comorbidities. The US Centers for Disease Control and Prevention (CDC) lists age over 65 years and residence in long-term care facilities as risk factors for severe COVID-19 [10], which is associated with increased hospital admissions and significantly increased mortality [10, 11]. Paradoxically, reducing the risk of SARS-CoV-2 transmission through isolation and social distance may increase the severity of anxiety symptoms, depressive symptoms, feelings of loneliness and perceived threat in society. The elderly, the sick, the less able and less resourceful are particularly susceptible to these symptoms [12].

### Grandparents as foster parents

Relatives become foster families most often out of necessity and a sense of duty in the face of the lack of responsibility on the part of other family members. This situation involves not only a desire to take care of a child, but also certain compulsion in the absence of other, better solutions in sight. Depending on the caregiver, two main types of kinship foster families can be distinguished. The first type are families where care is provided by grandparents; the second type are families with adult siblings in the role of caregivers. In the former case, the grandparents are the caregivers. Their children have limited parental authority, a situation that does not allow the natural closure of the family development cycle [13]. At a time when they should be acting only as grandparents, they have to fulfil parental roles. Grandparents most often have problems with keeping discipline, a situation that may be related both to a disturbed hierarchy of power in the family and to their age [14]. In Poland, 30% of caregivers in kinship families are older than 60 years. Decreased physical and psychological fitness limits educational activity, especially when it comes to assistance in learning, contact with teachers and development of child’s interests. The reduced financial possibilities of the family may also make it more difficult to satisfy the child’s needs [15]. Holtan et al. indicate that kinship foster parents are more likely to have a lower level of education than non-kinship foster carers [16]. This may contribute to the poorer material status, poorer access to health care and education for children, and a less healthy lifestyle. Reports from the US indicate that children from kinship foster families faced greater difficulties than children in non-kinship foster care. The caregivers of these children more often had a lower financial status, lower education, were unmarried and did not work. These families were more likely to experience food insecurity than non-kinship foster families [17]. Nevertheless, other studies highlight that placement in kinship foster care is more stable than placement in non-kinship foster care [18, 19].

Grandparents acting as parents are permissive, more tolerant and understanding; on the other hand, as representatives of a different generation, they are critical of how their grandchildren behave, being unable to understand their world, including fashion, music, ways of spending time, etc. [15]. Additional difficulties result from the life experiences of children who have been traumatised by the loss of parents, physical or psychological abuse or neglect. Behavioral and emotional problems of children placed in foster families are a serious challenge facing foster parents and social workers [18]. These difficulties can even take the form of children’s violence against their caregivers [20]. Australian study shows that a significant proportion of domestic violence involves kinship foster families. This violence concerns both carers and children from another family member, but there are also violent behaviors by children directed at carers [21]. Studies on this subject are rare and it is difficult to estimate the frequency of this phenomenon in other parts of the world [20]. When grandparents talk about problems plaguing their grandchildren’s biological family (parent’s mental illness, alcoholism, violence, etc.), they blame themselves and are unable to deal with their own emotions. Those grandchildren who witness these conversations or who become involved in the grandparents’ strife with their biological parents have great difficulty in achieving stability, above all in emotional terms. Establishing a foster family for a child sometimes does not result in changing the child’s place of residence and avoiding frequent contact with dysfunctional parents. The child continues to live together with their grandparents, who take care of them, often from the moment the child was born [3]. It is disturbing that in many cases it is impossible to avoid inheriting certain family dysfunctions, such as helplessness, irresponsibility, weak emotional bonds, addictions or violence, a fact that limits the chances of children in kinship foster care for development [22]. Sometimes this care is said to be only apparent, which is why it is often accompanied by the appointment of a probation officer as a form of support for the foster family and the possibility of permanent supervision of the child’s situation [23].

Research conducted in the UK during the COVID-19 pandemic revealed that kinship carers face a range of other challenges such as isolation due to their own illness or the illness of a family member (50%), a limiting long-term illness or disability (39%), disability children in care or special educational needs (37%), financial difficulties (25%) [24]. The above-mentioned difficulties may contribute to the deterioration of the psychosocial functioning of persons with kinship foster care. Research on this subject is necessary, as well as support from the state.

### The study of objectives and hypotheses

The aim of the study has been to identify the impact of the COVID-19 pandemic on the assessment of the performance of roles and responsibilities of people aged over 60 years who provide kinship foster care to their grandchildren in terms of their sense of self-efficacy and intensity of depressiveness. The following hypotheses were made about the study group:

1. The consequences of the COVID-19 pandemic have reduced the sense of self-efficacy;
2. The COVID-19 pandemic has exacerbated depressiveness.

## Methods

### Study procedure

The article has been written based on part of a study conducted within the research project entitled: “Identification of problems in medical, psychological and social aspects in people aged over 60 years who provide kinship foster care under a court decision to their grandchildren.”

The main assumptions of the global project were:

- identification of medical problems faced by foster carers aged over 60 who are related foster carers for their grandchildren;
- indication of social factors determining the profile of a kinship foster carer aged 60 plus (social origin, education, professional activity, material status, marital status, having biological offspring, fears and problems resulting from the role of a foster carer, dealing with difficult situations);
- identification of functional problems (mental, social and health) of kinship foster carers over 60 years of age in order to identify areas of deficits in fulfilling roles and responsibilities;
- assessment of the difficulties experienced by the respondents in terms of depression, self-efficacy, reaction to difficult, unforeseen situations or stress, coherence (sense of comprehensibility, sense of meaningfulness, sense of resourcefulness) and their relation to medical and psycho-social factors;
- determining the burden of kinship foster carers over 60 years old with caring for grandchildren of different ages;

an indication of the level and type of need for specialist support in fulfilling the role of a kinship foster carer.

The expected benefit of the undertaken research is exploring the issues of acting as a foster carer and at the same time a grandmother or grandfather. It is interesting that these people were the parents of children whose parental rights were limited or revoked by the court in adulthood. In these circumstances, it is reasonable to examine how now, as grandparents, they deal with the upbringing of their grandchildren. Factors such as reasons for limiting the parental rights of the biological parents were excluded.

The study was conducted in 2018–2019 in north-western Poland and repeated in late 2020 and early 2021. Two inclusion criteria were chosen for the study: 1) age − 60 years and older, 2) at least one of the foster caregivers was a biological grandparent. The first study was addressed to 189 kinship caregivers in the region of north-western Poland meeting the inclusion criterion. 78 caregivers agreed to take part in the study, i.e. 41.27% of those qualified. The study on the second date (during the pandemic) covered only 78 caregivers from the first phase. 54 families (representing 69.23% of the original sample) were contacted; 42 families were included in the study; in 8 families the child was removed from kinship foster care by court order; 2 families were unable to take part due to very poor health; 2 families refused to participate in the second part of the study. 40 out of 42 individuals completed the entire GSE and GDS questionnaires. Each participant was notified about the purpose and course of the study, as well as about the possibility to opt out at any phase without having to provide information about the reasons for this decision.

The first phase of the study was conducted by a trained worker in the place of residence of the foster family, while the second phase took place by telephone due to the COVID-19 sanitary restrictions and the interviews were conducted by the same worker. The study procedure received a positive opinion from the Bioethics Committee of the Pomeranian Medical University in Szczecin, registered under number KB-0012/166/03/18 on 29 March 2018.

### Measures

The study was conducted using the diagnostic survey method. In order to identify functional problems, the following measures were used:

1. Generalized Self-Efficacy Scale (GSE) - Polish adaptation by Ralf Schwarzer, Michael Jerusalem, ZygfrydJuczyński. It measures the strength of an individual’s general belief in their own efficacy in coping with difficult situations and obstacles. It contains 10 statements evaluated on a four-point scale (1 - no, 2 - rather no, 3 - rather yes, 4 - yes). The higher the score, the greater the sense of self-efficacy. The Cronbach’s alpha coefficient is 0.85 and the reliability of the scale assessed by test-retest by Juczyński after five weeks was 0.78 [25]. In our study the Cronbach’s alpha coefficient was 0.81 before the pandemic and 0.8 during the pandemic so the overall reliability of the questionnaires was achieved;
2. The Geriatric Depression Scale (GDS): the study used a 15-item version of the scale as the most commonly used screening tool for self-assessment of depression in the elderly. The GDS assesses the emotional state of the subject. A score in the range of 0–5 points is a normal result, 6–10 points indicate moderate depression, and 11–15 points indicate severe depression [26]. In our study, the reliability of the questionnaire was checked using the Cronbach’s alpha value, which was 0.66 before the pandemic and 0.81 for the survey taken during the pandemic. Similar to the GSE questionnaire, the overall reliability of the GDS questionnaires was achieved.
3. Own sociodemographic data questionnaire. Identification of medical problems was carried out on the basis of the nurse’s individual care card in Primary Health Care. This tool is standardized and constitutes Appendix No. 8 to Order No. 69/2007/DSOZ of September 25, 2007 of the President of the National Health Fund.

### Participants

**Fig. 1:**
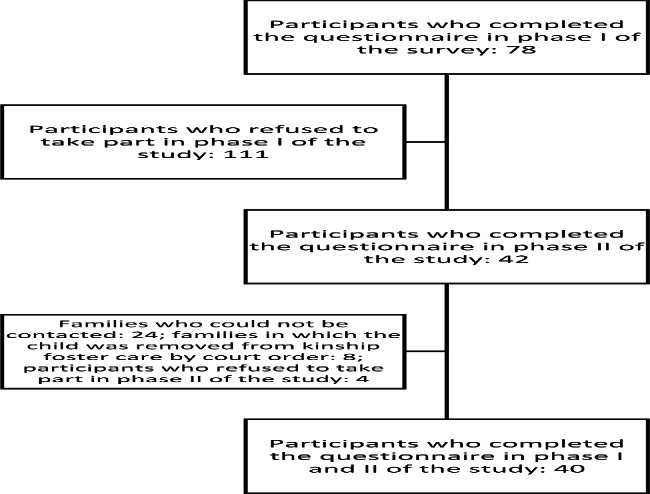
Participants included in the study

**Fig. 2:**
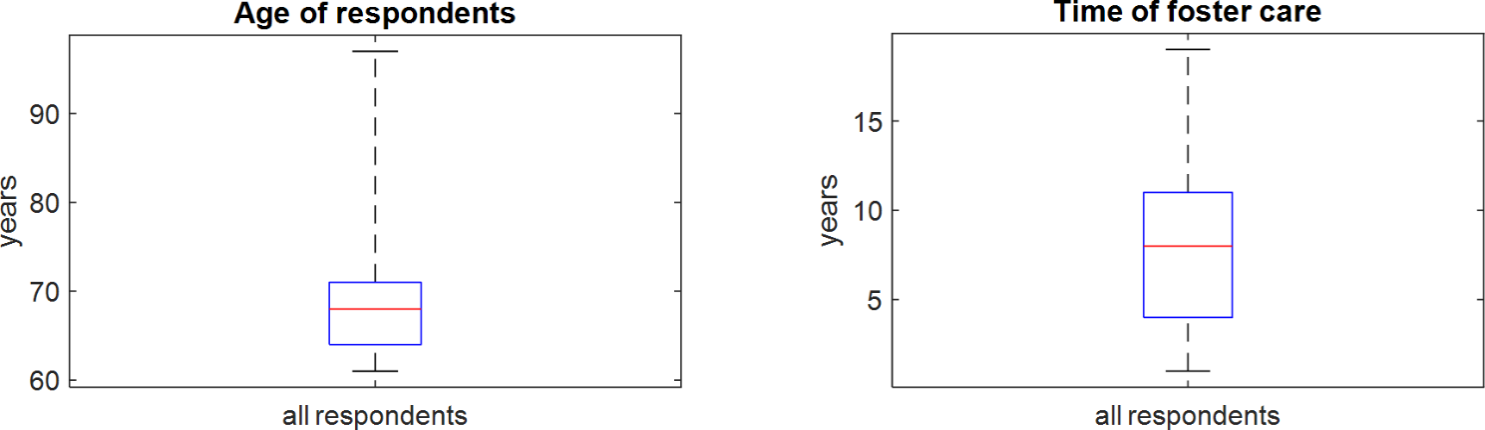
Median, upper and lower quartiles, minimum and maximum values of respondents’ age and duration of foster care for the whole group of the surveyed

A detailed description of study participant inclusion is shown in Fig. 1. The respondents come from north-western Poland. The average age of a person providing foster care is 68.78 years; the age range of all respondents is 61–97 years. The majority of participants in the survey were women, making up 87.5% of those surveyed. The health status was assessed as healthy by 60% of the people completing the questionnaire. The maximum duration of providing foster care by respondents was 19 years; the average duration was 8.25 years. The number of children in foster care ranged from 1 to 4 children, where the average was 1,425. Most respondents have vocational education (35%); 22.5% respondents have general education, and the least respondents have a university degree (10%). Box plots of the age of the surveyed and the duration of foster care are shown in Fig. 2. Detailed characteristics of the surveyed are shown in Table 1.

**Table 1:** Characteristics of the survey participants

Variable	Surveyed
N	40
Age (Mean ± SD) [in years]	68.78 ± 6.86
Age range (min – max) [in years]	61–97
Gender (F – female, M – male)	F − 35, M − 5
Good health	24
Mean duration of foster care (Mean ± SD) [in years]	8.25 ± 4.92
Mean number of children in foster care (Mean ± SD)	1.425 ± 0.75
Education (1 - primary, 2 - vocational, 3 - technical, 4 - general, 5 - higher)	1–8, 2–14, 3–5, 4–9, 5 − 4

### Statistical analysis

Quantitative data are given as mean ± standard deviation, median ± quartile deviation and as percentages. The Kolmogorov-Smirnov test showed that the distributions were not close to the normal distribution, so the Wilcoxon test was used to analyse the differences between the distributions. A significance level of 0.05 was assumed for all tests. The correlation between the results was examined using the Spearman’s correla-tion coefficient.

## Results

**Fig. 3:**
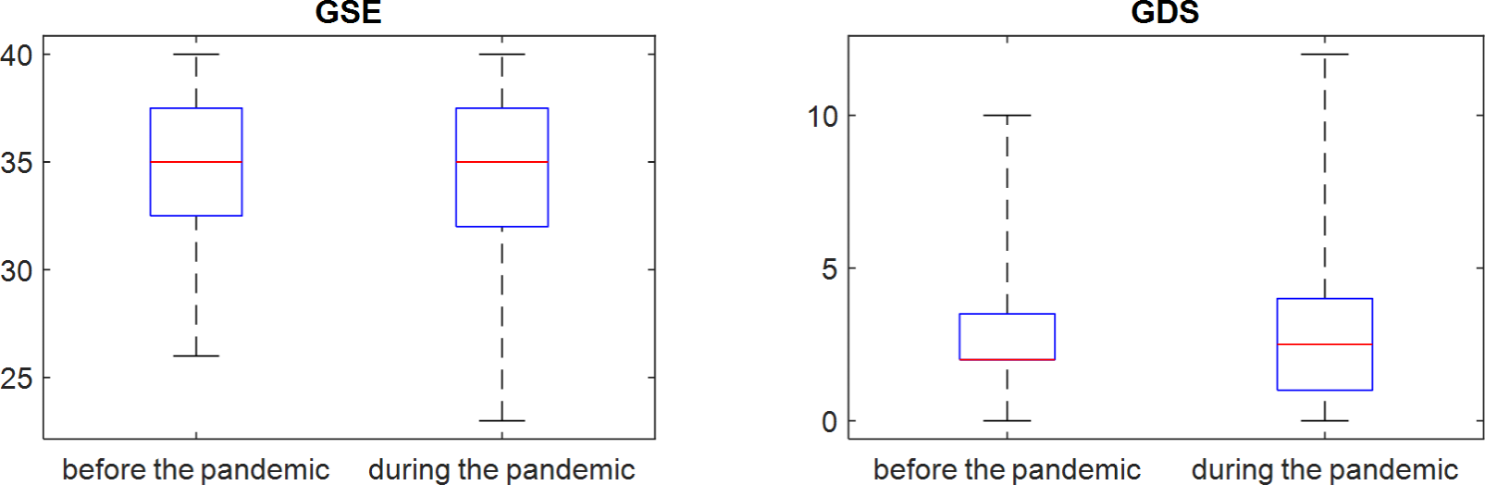
Median, upper and lower quartiles, and the minimum and maximum values for the results of the GSE and GDS surveys conducted before and during the pandemic

The results for the GSE survey in a box plot are shown in Fig. 3. It was shown with the Wilcoxon test that there were no statistically significant differences between the results obtained from the GSE survey before the pandemic and the GSE survey during the pandemic (p = 0.97), Table 2.

Using the Wilcoxon test, we investigated whether the values of the GSE survey con-ducted before and during the pandemic differed significantly among specific groups. People aged up to 65 years and over 65 years, those in good health and those in poor health, those with 1 child in foster care and those with more than 1 child were consid-ered, and the responses of those with the oldest child aged less than 10 years and over 10 years were examined. In each of these cases, statistical tests showed no significant differences between the results of the survey before the pandemic and during the pan-demic, Table 2.

The respondents’ sense of self-efficacy did not change significantly since the time be-fore the pandemic to the time of the second survey, i.e. during the pandemic.

**Table 2:** Comparison of the results of the GSE survey conducted before and during the pandemic, 40 respondents

			Wilcoxon test	
GSE	Mean ± SD	N	z-value	p
All respondents				
before the pandemic	34.75 ± 3.38	40	-0.038	0.97
during the pandemic	34.48 ± 3.86			
Respondents aged 65 years or less				
before the pandemic	35.29 ± 3.34	14	-0.9063	0.3648
during the pandemic	36.14 ± 3.13			
Respondents aged 66 years or more				
before the pandemic	34.46 ± 3.43	26	0.5400	0.5892
during the pandemic	33.57 ± 3.97			
Respondents with good health				
before the pandemic	34.67 ± 3.52	24	-0.698	0.485
during the pandemic	35.17 ± 3.37			
Respondents with poor health				
before the pandemic	34.88 ± 3.26	16	0.417	0.667
during the pandemic	33.44 ± 5.32			
One child in foster care				
before the pandemic	34.46 ± 3.57	28	-0.743	0.457
during the pandemic	34.71 ± 3.45			
More than one child in foster care				
before the pandemic	35.42 ± 2.91	12	0.787	0.431
during the pandemic	33.92 ± 4.81			
The oldest child in foster care aged 10 years or less				
before the pandemic	34.92 ± 3.12	13	-0.433	0.665
during the pandemic	35 ± 3.72			
The oldest child in foster care, aged 11 or more				
before the pandemic	34.67 ± 3.55	27	0.189	0.85
during the pandemic	34.22 ± 3.97			

The median, lower and upper quartiles, and minimum and maximum values for the results of the GDS survey conducted before and during the pandemic are shown in Fig. 3. Based on the Wilcoxon test, it cannot be concluded that there are statistically significant differences between the respondents’ answers to the GDS survey beforeand during the pandemic (p = 0.94), Table 3. The Wilcoxon test was also performed for subgroups of individuals (as for the GSE) selected from among all the respondents. In one case, for those with the oldest child in foster care aged 10 years or less, the Wilcoxon test showed a statistically significant difference between responses in the two periods. In this case, the mean value of the GDS index is 3.54 in the survey before the pandemic and 2.77 in the survey during the pandemic. This mean that it has decreased during this period but remains low. The survey thus shows that the COVID-19 pandemic has not exacerbated depressiveness among respondents.

**Table 3:** Comparison of the results of the GDS survey conducted before and during the pandemic, 40 respondents

			Wilcoxon test	
GDS	Mean ± SD	N	z-value	p
All respondents				
before the pandemic	2.88 ± 2.13	40	0.07	0.94
during the pandemic	3.05 ± 2.89			
Respondents aged 65 years or less				
before the pandemic	2.07 ± 1.21	14	1.4606	0.1441
during the pandemic	1.57 ± 1.5			
Respondents aged 66 years or more				
before the pandemic	3.31 ± 2.40	26	-0.8163	0.4143
during the pandemic	3.85 ± 3.16			
Respondents with good health				
before the pandemic	2.38 ± 1.79	24	0.996	0.319
during the pandemic	2.13 ± 2.07			
Respondents with poor health				
before the pandemic	3.63 ± 2.42	16	-0.808	0.419
during the pandemic	4.44 ± 3.43			
One child in foster care				
before the pandemic	2.82 ± 1.91	28	0.965	0.334
during the pandemic	2.61 ± 2.59			
More than one child in foster care				
before the pandemic	3 ± 2.66	12	-1.467	0.142
during the pandemic	4.08 ± 3.4			
The oldest child in foster care aged 10 years or less				
before the pandemic	3.54 ± 2.67	13	2.15	0.0313
during the pandemic	2.77 ± 2.59			
The oldest child in foster care, aged 11 or more				
before the pandemic	2.56 ± 1.78	27	-1.246	0.213
during the pandemic	3.19 ± 3.06			

The analysis of the Spearman’s correlation between the GSE and GDS indices shows that the increase in the GSE index was associated with the decrease in the GDS index.. The correlation coefficient is -0.46 (p = 0.003) for the data in the first period before the pandemic and − 0.43 (p = 0.006) for the results obtained during the pandemic. The correlation coefficient for the GSE data collected before the pandemic and the GDS results during the pandemic was − 0.37 (p = 0.02). In all three cases the correlations are statistically significant; the p-value w is < 0.05.

Table 4 shows the distribution of the 15-item GDS scale by 0–5 points, 6–10 points and 11–15 points. The data indicate that the number of individuals with a higher GDS score increased during the pandemic. Compared to the study before the pandemic, in the range of 6–10, the number of people increased to 7, while in the range of 11–15, the result was obtained for one person. However, the statistical test presented in Table 3 shows that these differences are not statistically significant.

**Table 4:** Number of subjects scoring in a given range for the GDS questionnaire, the test performed before and during the pandemic

GDS score	N before the pandemic	N during the pandemic
0–5	37	32
6–10	3	7
11–15	0	1

## Discussion

It is worth noting that the study participants both before and during the pandemic rated their self-efficacy highly, obtaining an average score of about 34 points out of 40 possible in both phase I and II. The fact that the scores before and during the pandemic were remarkably similar may indicate that the sense of self-efficacy as measured by the GSE is not affected by external factors such as the COVID-19 pandemic.

The GSE scores are higher than those found in groups of persons who do not acts as foster parents of their own grandchildren. E.g. in his study, Juczyński indicates that people in the age range of 30–50 years scored 27.32 on average, less than in our own study [27]. Higher results were obtained neither among athletes [28] nor among nurses completing postgraduate education [29].

The results of the research are all the more interesting because the introduction of the article indicates many stressors, but an in-depth analysis of the issues related to kinship foster care indicates a number of protective aspects.

The obtained results may be explained by the fact that people aged over 60 years who adopted their own grandchildren have a more difficult life experience and, due to their role, often have had to cope with challenging situations. With this awareness, the reality of the pandemic has neither affected their self-efficacy score nor led to the development of depressive symptoms. These results are consistent with those obtained in other studies comparing the intensity of depressive symptoms among young adults, middle-aged people and the elderly during the pandemic. The symptoms were least severe among the elderly [30].

It is worth noting that, due to multiple caring responsibilities, the study group in question experienced no isolation or loneliness during the pandemic, a factor that may have protected them against developing depressiveness. Furthermore, the study participants were not stressed about losing their job, probably due to being pensioners and professionally inactive.

It is important for the study results to note that kinship foster families are supported and supervised by social welfare centres and family courts, and that foster caregivers are subject to a two-stage process of qualification and assessment for prospective kinship foster families. The first stage consists in submitting the documentation required by the court, and the second phase consists in analysing the documentation and issuing an opinion on meeting the required conditions and having predispositions to provide foster care.

The legal procedure for qualifying candidates for kinship foster carers can therefore be considered one of the factors protecting people taking on this responsibility, because each candidate is verified in terms of ability to fulfill caring roles.

A kinship foster family receives a monthly payment to cover the costs of maintaining a child in a foster family and may benefit from counselling and training.

Institutional support of the kinship forms of foster care, good preparation for this role and awareness of this role may be factors influencing the results of the research.

In conclusion, it is probable be that the study participants, having relatively high GSE scores already before the pandemic, coped with the pandemic without showing any depressive symptoms at the time. Moreover, their difficult life situation paradoxically has protected them from the harmful effects of the pandemic. The responsibility of being a foster carer, especially during the COVID 19 pandemic, could become a motivating factor resulting in a high level of self-efficacy.

### Limitations

A significant limitation of the research is the small sample size of N = 40, which makes it impossible to generalize the results. The study is also limited by its self-report nature, which was caused by the pandemic and sanitary restrictions.

## Conclusions

1. The sense of self-efficacy among the study subjects has not changed significantly when comparing the time before the pandemic with the time during the pandemic.
2. The COVID-19 pandemic has not exacerbated depressiveness among the respondents.
3. Both before and during the pandemic, the increase in depressiveness was associated with the decrease in self-efficacy.

## Data Availability

The datasets used and/or analysed during the current study are available from the corresponding author on reasonable request.

## List of Abbreviations

LLD: Late-life depression
WHO: World Health Organization
SARS-CoV-2: Severe acute respiratory syndrome coronavirus
GSE: Generalized Self-Efficacy Scale
GDS: The Geriatric Depression Scale.
